# A community-based intervention in middle schools to improve HPV vaccination and cervical cancer screening in Japan

**DOI:** 10.1186/s12930-014-0013-0

**Published:** 2014-11-18

**Authors:** Tomoko Ito, Remi Takenoshita, Keiichiro Narumoto, Melissa Plegue, Ananda Sen, Benjamin Franklin Crabtree, Michael Derwin Fetters

**Affiliations:** Shiga Center for Family Medicine, 1825 Yuge, Ryuo, Gamo District, Shiga Prefecture, 520-2501 Japan; Shizuoka Family Medicine Program, Shizuoka, Japan; Department of Obstetrics, Gynecology and Family Medicine, Hamamatsu University, School of Medicine, Hamamatsu, Shizuoka Japan; Department of Family Medicine, University of Michigan, Ann Arbor, Michigan USA; Department of Family Medicine and Community Health, Research Division, Rutgers Robert Wood Johnson Medical School, Piscataway, New Jersey USA; Mori Machi Family Medicine Clinic, 387-1 Kusagaya, Mori, Shuchi District, Shizuoka Prefecture, 437-0214 Japan

**Keywords:** Early detection of cancer, Japan, Papillomavirus vaccines, Vaccination, Intervention

## Abstract

**Aim:**

Japan has low rates of cervical cancer screening and Human papilloma virus (HPV) vaccination. This research examines the effectiveness of a family medicine resident-led, intervention in increasing knowledge about HPV and cervical cancer in middle school-girls and increasing knowledge and intention to have cervical cancer screening in their mothers.

**Methods:**

We utilized a pre-test/post-test intervention design in three rural middle schools with 7^th^ grade middle school-girls and their mothers. A school-based activity educated girls about HPV and cervical cancer. A home-based activity utilized a homework assignment for girls and their mothers. Pre/post intervention surveys were completed by the girls and their mothers. Major outcomes included changes in knowledge among girls and mothers and barriers to be screened for cervical cancer among mothers.

**Results:**

Sixty-five students and sixty-three mothers completed the study. Two out five mothers were not in compliance with current screening recommendations. Identified barriers included: embarrassment (79%), poor access (56%), fear of having cancer (52%), and cervical cancer screening being an unknown procedure (46%). Forty-four percent of mothers deemed their daughters to be at risk for cervical cancer. Trusted sources of information included: doctors (97%), newspapers/television (89%), government (79%), the Internet (78%), and friends (62%). Student knowledge scores (7-point scale) improved significantly from pre- to post-intervention (4.8 vs. 5.9, p < 0.001). Knowledge scores (14-point scale) among mothers also significantly improved (11.7 vs. 12.0, p = 0.024).

**Conclusions:**

These data suggest a community-based intervention on a sensitive topic by family medicine residents can be implemented in middle schools, can improve school-girls’ knowledge about HPV and cervical cancer, and can reach their mothers. Additional research could examine whether those intending to be screened receive screening and how to reach women who still resist screening.

## Summary of implications of the research/article for practicing GP’s

This research illustrates that a community-based intervention featuring a lecture by family physicians to middle school-girls, followed by a homework assignment for the girls and mothers, can increase knowledge about HPV vaccination and cervical cancer screening. About two of five mothers in this rural area were not in compliance with cervical cancer screening. Reported barriers to screening included: embarrassment, poor access, fear of having cancer, and cervical cancer screening being an unknown procedure. While these girls and mothers understand that HPV infection can cause cancer, confusion persisted about whether all forms of HPV infection are linked to cervical cancer. The intervention did not increase substantively the number of mothers intending to be screened; this is likely due to high rates of intention to be screened at baseline. As virtually all mothers consider physicians a trustworthy information source, physicians should actively encourage daughter and mother participation in these preventive services.

## Introduction

Cervical cancer is the second most common cancer among women between the ages of 20–49 in Japan [1]. Japan had a national cervical cancer screening program from 1982 to 1998 targeting women 40 years of age and older; however, this national program ended when the responsibility was transferred to local governments. The current cervical cancer screening recommendation in Japan is to screen women 20 years of age and older every two years [2]. Since 2009, the Japanese government has offered a free cervical cancer screening coupon to women at the ages of 20, 25, 30, 35, and 40 [3]. Despite these recommendations and incentives, Japan has the lowest rate of cervical cancer screening among developed countries [4].

Despite an initial start with a compulsory vaccination program, Japan differs from other developed countries in the adoption of HPV vaccination. Human Papilloma Virus (HPV) vaccination has been shown to be effective for preventing cervical cancer and began in 2009 in Japan. A compulsory program to have HPV vaccinations began in Japan in April of 2013; however, case reports emerged suggesting severe side effects of HPV vaccine. Hence, the Japanese government withdrew the compulsory program in June, 2013. The Japan Times [5] reported a total of 8.29 million people had received HPV vaccines through December 2012. According to a Ministry of Health, Labour and Welfare (MHLW) panel, 1,925 cases of side effects were reported through the end of December 2012. In the MHLW report from July, 2014, the rate of very serious side effects per one million HPV inoculations included: anaphylaxis - one case, Guillan-Barre syndrome - 0.6 cases, acute disseminated encephalomyelitis (ADEM) - 0.4 cases, though the relationship between these symptoms and vaccination were not proven. In all, 20 cases per 1 million were rated as serious cases of pains or body convulsions, pains in joints or difficulty in walking [6]. This is less than the 26.0 serious cases per million inoculations of Japanese encephalitis vaccine [5]. With regard to the burden of cervical cancer in Japan, in a ten-year period, about 16,000-28,000 women per year are diagnosed with cervical cancer and about 2,400-2,700 of them die as a result [7-9].

Educational efforts by health professionals have the potential to enhance knowledge among adolescents and adult women about cervical cancer, the purpose of screening, and the value of prevention through HPV vaccination [3,10]. Given their role in “womb-to-tomb” care, family physicians are well placed in the community for playing an influential role in promoting HPV vaccination and cervical cancer screening. The purpose of this research was to assess the feasibility of a family medicine resident-led, school-based educational intervention to increase knowledge of cervical cancer and the role of HPV vaccinations in middle school-girls while indirectly increasing knowledge and cervical cancer screening intentions in their mothers.

## Materials and methods

### Design, setting and participants

**Figure 1:**
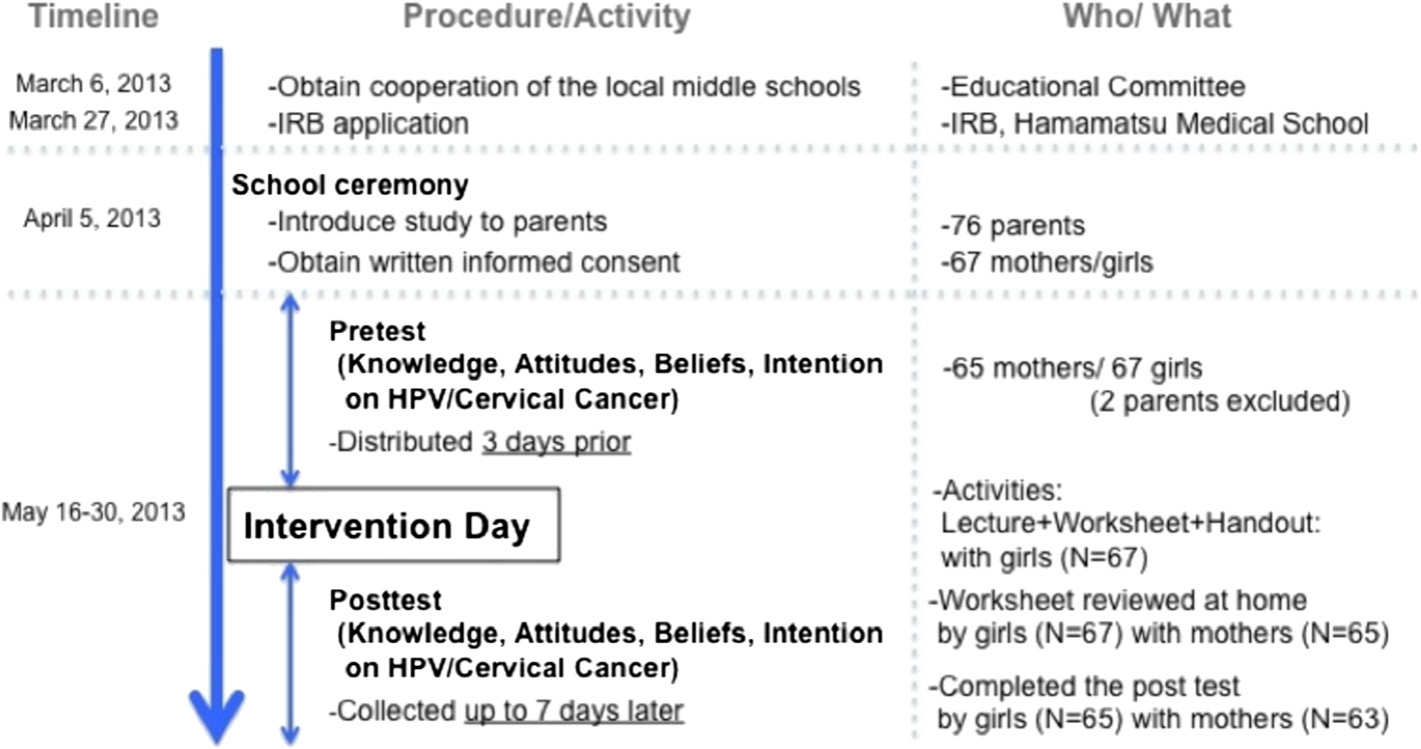
**Study design.**

We utilized a pre-test/post-test intervention design (Figure 1). The study took place in three middle schools in the rural town of Shizuoka Prefecture, Japan in April and May, 2013. Participants included first-year female middle school students (7^th^ grade equivalent) from the three schools and their mothers. All female students and their mothers were eligible for inclusion; there were no exclusion criteria. This project was approved by the Hamamatsu Medical School Institutional Review Board.

### Intervention

The project involved a two-part intervention with both school-based and home-based components. The school-based component lasted approximately one hour, and was comprised of a 20-minute slide presentation by two female family physicians (TI and RT) about HPV and cervical cancer, and a 9-item worksheet filled out by middle school-girls during the presentation. It was carried out using an interactive-lecture format in a school classroom. The content was developed to address three fundamental topics: 1) the high prevalence and incidence of cervical cancer among young women, 2) cervical cancer is preventable and 3) how to prevent cervical cancer. The school-based component was reviewed with teacher representatives of the three participating middle schools. Any content felt by the teachers to have sexual connotations was not acceptable and removed. The school teachers agreed to the final content. The home-based component centered on a required homework assignment in which the girls reviewed the worksheet they had completed with their mothers.

### Data collection

Pre-/post-surveys about HPV and cervical cancer screening were administered to assess knowledge of HPV and cervical cancer among the middle school-girls. The investigators distributed the pre-intervention survey three days before the school-based exercise with the girls. The post-intervention survey was distributed after the school-based exercise taken by the girls, and the post-intervention surveys were collected within the following seven days.

This survey was developed using questions from previously published surveys [11-19] and included 9 true/false questions that required 5–10 minutes to complete. A similar instrument that differed by having more questions was administered to the mothers that assessed knowledge, beliefs about HPV and cervical cancer, as well as future intentions to be screened for cervical cancer. The instrument for mothers included 20 items. This survey involved true/false questions (including the same questions asked of the daughters), as well as additional questions that were specific to the mothers or deemed not appropriate for students by the school (ie., whether HPV is sexually transmitted, whether having the vaccine precludes the need for screening, and whether cervical cancer screening should be done regularly). Additional questions were posed to mothers using a four-point Likert scale addressing prevention of cervical cancer, their risks of cervical cancer for their daughters in the future, barriers to cervical cancer screening, trustworthy sources of information, and appropriate times to educate daughters about cervical cancer screening. This instrument required mothers about 15 to 20 minutes to complete. Mothers also completed five demographics items on age, highest educational level, personal and family experience with cervical cancer, and history of cervical cancer screening administered pre-intervention only. Both student and mother instruments targeted a 6^th^ grade reading level.

### Data analysis

Descriptive statistics were calculated for demographic variables filled out by the mothers at the baseline survey. The major outcomes included changes in student knowledge of HPV and cervical cancer pre- and post-intervention, mothers’ knowledge, attitudes and beliefs, mothers’ intentions to obtain cervical cancer screening, and differences between mothers compliant with cervical cancer screening recommendations and those not compliant. Sixty-three mothers completed both the pre- and post-intervention survey (Table 1). Two mother-daughter pairs did not answer any questions on the post questionnaire and were not included in the analyses. For some instruments, participants did not answer all questions. Such unanswered questions were treated as missing values and the results reported are based on valid responses, except as indicated below for the knowledge questions.

**Table 1:** **Mother demographics**

**N = 63**	**Compliant (n = 39)**	**Non-compliant (n = 25)**	**Overall N (%)**
**Age, p-value = 0.803**			
<=30	1 (2.6)	0 (0.0)	1 (1.6)
31-35	3 (7.9)	4 (16.0)	7 (11.1)
36-40	10 (26.3)	7 (28.0)	17 (27.0)
41-45	19 (50.0)	10 (40.0)	29 (46.0)
> = 46	5 (13.2)	4 (16.0)	9 (14.3)
**Education, p value = 0.332**			
Junior High School	2 (5.3)	0 (0.0)	2 (3.2)
High School	13 (34.2)	14 (56.0)	27 (42.9)
College/Special School	19 (50.0)	9 (36.0)	28 (44.4)
University	4 (10.5)	2 (8.0)	6 (9.5)
**Cervical Cancer History [n = 58], p value = 1.00**			
Herself	1 (2.9)	0 (0.0)	1 (1.7)
Family/Relatives	1 (2.9)	0 (0.0)	1 (1.7)
Friends	2 (5.7)	2 (8.7)	4 (6.9)
None/Don’t Know	31 (88.6)	21 (91.3)	52 (89.7)

Knowledge scores for students were computed by summing together the number of correct responses on seven applicable knowledge questions asked at both pre- and post-intervention. In cases where students missed answering a question (one case at pre- and one post-intervention), missing responses were treated as ‘incorrect’ responses. Two questionnaires filled out by fathers were dropped from the analyses since the questions were designed for women. The two girls whose fathers had completed the parent survey were included in the analysis of student scores. All 67 students had scores for the pre-survey and at post there were 65 valid scores (2 students did not complete any of the post questionnaire). Scores could range from 0 to 7 based on responses to the 7 knowledge questions. Average scores were compared for the 65 students who completed both surveys using a paired t-test.

Mothers’ knowledge scores were computed by summing together the number of correct responses on 14 applicable knowledge questions asked at both pre- and post-intervention. As with students, a missing response by any of the mothers when other questions had been answered was considered incorrect. Knowledge scores could range between 0 and 14. The item measuring intention to be screened in the future, “Do you intend to be screened for cervical cancer?” originally had responses of “no,” “yes, in this year” and “yes, in 2–3 years.” This was dichotomized into “no” or “yes.”

Regarding two change-in-beliefs questions asking about mothers’ trust of information from multiple sources and appropriate timing of education about cervical cancer education, the categories (strongly disagree, disagree, agree and strongly agree) were collapsed into two categories (agree vs. disagree). The responses to these questions from mothers between pre- and post- were compared using the McNemar test.

## Results

### Demographics

Sixty-five female 7^th^ grade students completed the study and were either 12 or 13 years of age. Most mothers were over the age of 40 (60%) and all but 2 had graduated from high school. Regarding personal experience with cervical cancer, only one of the mothers had a personal history of cervical cancer, while five had a relative or friend who had had cervical cancer. At baseline 19 mothers reported that they had never been screened for cervical cancer, while another 6 indicated that they had not been screened in more than 3 years. Thus, 25 (40%) mothers were not compliant with screening recommendations at baseline. Regarding perception of their daughters’ vulnerability to getting cervical cancer in the future, 28 (44%) respondents agreed or strongly agreed that their daughters were vulnerable to future cervical cancer. There was no relationship between agreeing/disagreeing with this statement and maternal age, education, cervical cancer history or intention to be screened pre-intervention.

### Daughters’ and mothers’ knowledge about HPV and cervical cancer

**Figure 2:**
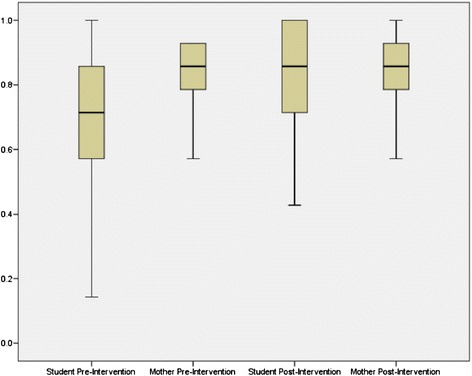
Comparison of student and mother knowledge scores pre- and post- intervention*. *Since students and mothers answered a different number of knowledge questions, student scores could range from 0 to 7, while mother scores could range from 0 to 14. Comparison on the same scale was made by dividing the score by the maximum possible (7 for students and 14 for mothers).

Figure 2 provides a comparison of student and mother knowledge scores. Average knowledge scores for students on a seven-point scale were significantly higher from before to after the intervention (4.8 vs. 5.9, p-value < 0.001). Similarly, average scores for mothers on a 14-point scale for the 63 mothers who completed both surveys were significantly higher from before to after the intervention (11.7 vs. 12.0, p-value = 0.024).

The relationship between change in score for both mother and daughter was investigated by categorizing the change for both groups as increasing, staying the same or decreasing. Cross-tabulation was used to examine the relationship between corresponding mother and daughter score changes. A chi-square test was not significant (Fisher’s Exact p-value = 0.664), indicating that a change in score for students from pre- to post- was independent of change in score for mothers, i.e., increase in students’ knowledge scores doesn’t correspond to an increase in mothers’ knowledge scores.

As shown in Table 2, responses to specific knowledge questions improved for most items; however, unexpectedly, both student and mother understanding that all HPV infections do not lead to cervical cancer decreased. Otherwise, for students, increase in knowledge scores were seen on all other questions with greatest gains in understanding that HPV causes cervical cancer (44% improvement), cervical cancer is asymptomatic at an early stage (32% improvement), and cervical cancer is preventable (25% improvement). Mother knowledge scores were high at baseline, and increases in knowledge were modest, though a clinically notable difference was a 14 percentage point gain for understanding that safe sexual practices can prevent cancer. When comparing knowledge scores among mothers compliant with cervical cancer screening recommendations and among mothers not compliant, there were no significant differences.

**Table 2:** **Correct knowledge about HPV vaccine and cervical cancer responses of students and mothers**

	**Students, N = 65**		**Mothers, N = 63**	
	**Pre**	**Post**	**Pre**	**Post**
Cervical cancer is caused by HPV infection?	46%	100%	86%	97%
Cervical cancer is genetic?	51%	63%	71%	81%
All HPV infections lead to cervical cancer?	75%	49%	89%	76%
Cervical cancer is decreasing in recent years?	83%	92%	100%	98%
Cervical cancer is preventable?	72%	97%	70%	78%
Cervical cancer is asymptomatic usually in early stage?	65%	97%	98%	98%
Early detection of cervical cancer might save one’s life?	85%	92%	95%	97%
HPV is sexually transmitted?	NA	NA	91%	97%
If vaccinated, you don’t need to be screened?	NA	NA	98%	97%
Screening test should be done regularly?	NA	NA	92%	95%
HPV vaccination can prevent cervical cancer	NA	NA	95%	95%
Safe sex (steady partner, use of condoms) can prevent cervical cancer	NA	NA	75%	89%
Pap test can prevent cervical cancer	NA	NA	94%	95%
Healthy lifestyle (ie., regular exercise, disciplined lifestyle, healthy diet) can prevent cervical cancer	NA	NA	11%	11%

NA = Not asked as deemed to be too sensitive and unacceptable by the local middle schools.

### Mothers’ beliefs about barriers to cervical cancer screening

Mothers also responded to questions about barriers to cervical cancer screening and prevention of cervical cancer. At the baseline assessment, many mothers identified barriers to cervical cancer screening including: embarrassment (79%), poor access (56%), fear of having cancer (52%), and cervical cancer screening being an unknown procedure (46%). Among mothers who were in compliance and mothers out of compliance with cervical cancer screening recommendations, there were differences in their responses on the importance of cervical cancer screening being an unknown procedure, with 72% of non-compliant mothers agreeing and only 29% of compliant mothers agreeing (p-value < 0.001).

### Mothers’ beliefs about trustworthy sources of information and timing of education about HPV and cervical cancer screening

Mothers answered questions about sources of trusted information and beliefs about when it is appropriate to educate women at baseline assessment. Regarding sources of trusted information about cervical cancer, the percent indicating strong agreement or agreement for five sources was: doctors (97%), newspapers/television (89%), government (79%), the Internet (78%), and friends (62%). Mothers also were asked about the appropriate timing of education about cervical cancer, and at baseline, the percent indicating strong agreement or agreement for four sources was: beginning of junior high (83%), end of junior high (79%), high school (65%) and college or later (52%). When compared pre- and post- intervention using McNemar’s test, there were no differences for either trusted source of information or beliefs, an indication that the intervention did not have an effect on these factors.

### Impact of intervention on intention to receive cervical cancer screening among those not compliant with screening guidelines

Twenty-five mothers (40%), including 19 who had reported no history of cervical cancer screening and six who reported no screening in over 3 years, were not in compliance with Japanese screening recommendations. In the subset of 23 mothers who answered both the pre- and post-intervention survey, five women who had not indicated an intention to be screened at baseline, changed and indicated intention to be screened after the intervention. A McNemar test indicated that the intervention did not significantly promote intention to receive cervical cancer screening on those out of compliance, though sample size is small so ability to make inferences is limited. A chi-squared test indicated a significant relationship with those who had been screened in the past being more likely to intend on being screened again (100.0% vs. 65.2%, p < 0.001).

## Discussion

These data provide information about the nature of knowledge about HPV vaccination and cervical cancer risk in one rural community. These findings illustrate that two out of five mothers in this rural area were not in compliance with cervical cancer screening. Identified barriers to screening included: embarrassment, poor access, fear of having cancer, and cervical cancer screening being an unknown procedure. As previous screening predicts future intention to be screened, the women with greatest need are those who are not currently compliant. Close to half of mothers (44%) feel that their daughters are at risk for cervical cancer in the future. For their education, mothers identify as particularly trusted sources of information their doctors and media/newspapers.

Student and mother knowledge scores improved significantly from baseline to post-intervention, indicating that in the short term this school-based intervention was effective for promoting knowledge about HPV vaccination and cervical cancer screening for 7^th^ grade girls. The magnitude of improvement was much greater for the students than mothers, attributable to the high baseline knowledge scores. It was not possible to assess long-term knowledge retention, nor whether the intervention actually impacted the rate of HPV vaccinations among these middle school-girls, nor the rate of cervical cancer screening of their mothers.

Despite high knowledge scores at baseline, 40% of the mothers were not in compliance with cervical cancer screening. This suggests that there are other factors besides knowledge that impact these women’s decision making about whether to receive cervical cancer screening. Previous research suggests factors that may prove to be barriers to cervical cancer screening. Fetters et al. found that 12 of 19 Japanese women receiving care in a U.S. clinic approved of the U.S. style of the pelvic examination and they valued such measures as using a private room, covering the perineum with a sheet and explaining the procedures being used [20]. This contrasts with the typical style of examination in Japan where a woman receives pelvic examinations lying on an examination table in a stall with a curtain placed at the waist for privacy even though her pelvis is exposed openly to staff [20]. In an opinion paper, Konno et al. mentioned that in order to make public health measures of cervical cancer effective, education, environment and enforcement are very important, but that the Japanese cervical cancer screening environment gives the patients no control and often no privacy [3]. In related research from Taiwan, Wu found barriers to breast cancer screening to include: 1) excuses (no time, forgot, cumbersome and lazy), 2) no need for screening, 3) modesty, 4) discomfort, 5) logistics, 6) lack of information/knowledge, and 7) fear of finding cancer [11]. In their report about barriers to HPV vaccination in Asia Oceania, Garland et al. raise the barrier of reluctance to discuss issues around sex [11]. This barrier clearly applies in Japan where medical students have no routine exposure to discussions on sexuality. Few family medicine training programs in Japan even offer women’s health training [21]. Few practicing primary care physicians provide contraceptive or prenatal [22,23]. Reluctance to have screening may also reflect a sense of vulnerability, or unpleasant, negative experiences, e.g., discomfort, poor explanations in the past [3,20]. Future research should explore the potential barriers to, and benefits of, women seeking care for women’s health issues with family physicians.

There are a number of limitations to this study. While the educational intervention content was appropriate for improving student knowledge, it may be insufficient for the educational needs of their mothers. While there was meaningful increase in the number of women who intended to be screened after the intervention, it is not clear whether the intervention was actually linked to the mothers’ intentions to be screened. A number of mothers reported they had never been screened for cervical cancer. As obtaining a Pap smear is routine practice with all pregnant women, it is likely that those reporting that they had never been screened were unaware of that they had been screened when pregnant with their daughters-interpreting them as non-compliant is still a reasonable position. Finally, the current survey was unable to discern reasons for why women continue to resist cervical cancer screening despite apparently having good knowledge about cervical cancer and HPV. Future research could explore such reasons with the aid of depth interviews of mothers and daughters from both the “changed-behavior group” and the “unchanged-behavior group.”

A follow-up survey will be needed to determine if intention to receive cervical cancer screening translates into care-seeking behavior. The current research suggests this relatively inexpensive, community-based intervention provides a novel strategy for reaching individuals in the community who are not necessarily accessing the health care system. If further research on actual behavior is positive, such community-based interventions could be used as a tool for increasing HPV vaccination and cervical cancer screening throughout Japan.
